# Effect of a high vs. standard dose of vitamin D3 supplementation on bone metabolism and kidney function in children with chronic kidney disease

**DOI:** 10.3389/fped.2022.990724

**Published:** 2022-11-04

**Authors:** Zhiqiang Feng, Kunna Lu, Yan Ma, Feng Liu, Xinhuan Zhang, Hongxiang Li, Yan Fu

**Affiliations:** ^1^Department of Pediatric Surgery, Taian Maternal and Child Health-Care Hospital, Tai'an, China; ^2^Department of Endocrinology, The Second Affiliated Hospital of Shandong First Medical University, Tai'an, China; ^3^Department of Surgery, Taian City Central Hospital, Tai'an, China; ^4^Department of Neonatal Intensive Care Unit, The Second Affiliated Hospital of Shandong First Medical University, Tai'an, China; ^5^The Second Affiliated Hospital of Shandong First Medical University, Tai'an, China

**Keywords:** chronic kidney disease, vitamin D, bone metabolism, bone strength, kidney function

## Abstract

We investigated the effects of high- vs. standard-dose vitamin D supplementation on kidney function and bone metabolism in children with chronic kidney disease (CKD). Children were randomized to receive one of two formulations: 75 participants received 2,000 IU/D of oral supplementation of vitamin D, while 75 participants received 400 IU/d for a minimum of 4 months. We investigated the effects of vitamin D supplementation on kidney-related indicators and bone metabolism-related indicators at different doses. A total of 158 participants were screened, among whom 150 met the inclusion criteria. The indicators of chronic kidney disease such as eGFR and serum uric acid were negatively correlated with the 25(OH)D level and BMD. Serum 25(OH)D and osteocalcin levels were positively correlated with spine BMD. The standard dose of vitamin D can improve the serum uric acid level, but high doses of vitamin D supplementation had no significant effect on the serum uric acid level. High doses of vitamin D supplementation can also improve the alkaline phosphatase level. When comparing the results of different doses of vitamin D supplementation, it was found that high-dose vitamin D supplementation did not improve bone density in the spine and femur neck relative to the standard dose of vitamin D but improved hypocalcemia and N-terminal propeptide of the human procollagen type I (PINP) level. Among the children with clinical kidney disease, high-dose vitamin D treatment for 4 months resulted in statistically significant improvement in kidney function but no significant difference in bone metabolism compared with the standard-dose vitamin D treatment.

## Introduction

Chronic kidney disease (CKD) is a disease that seriously affects public health (1). The incidence and prevalence of chronic kidney disease are increasing in children. In children, chronic kidney disease increases mortality (2, 3). CKD has a significant impact on a variety of factors, including growth, cognition and behavior, and cardiovascular health (4–7), as well as a significant impact on the quality of life of children (8). The burden of chronic kidney disease (CKD) and its treatment may severely limit the ability of children with CKD to do daily tasks and participate in family, school, sports, and recreational activities (9). Chronic kidney disease has a significant effect on the absorption of calcium and phosphorus, which, in turn, affects bone system health (10). Therefore, the label “kidney-induced osteoporosis” has been proposed. CKD is also associated with an increased risk of osteoporosis fractures (11). However, studies have also found that the relationship between osteoporosis and CKD is not very close (12).

Calcium, phosphate, and the vitamin D hormonal system are key players in chronic kidney disease–mineral and bone disorder (CKD-MBD). Vitamin D plays an important role in kidney and bone health. Vitamin D deficiency is very common among patients with CKD and has a significant impact on the progression of CKD. In addition, vitamin D principally influences skeletal mineralization through the regulation of intestinal calcium absorption (13). However, meta-analyses of trials show vitamin D has no effects on bone density or fracture risk when the baseline 25-hydroxyvitamin D is >40 nmol/L, and a daily dose of 400 to 800 IU of vitamin D3 is often adequate to correct such deficiency (14). A randomized clinical trial found that it is not helpful for high-dose vitamin D supplementation for bone health (15). The effect of different doses of vitamin D3 on bone metabolism in children with chronic kidney disease is not yet known.

Therefore, it is important to clarify the effects of different doses of vitamin D on the skeletal system and renal function. Therefore, the purpose of this study was to analyze the effects of high-dose and standard-dose VitD3 supplements on kidney function and bone metabolism in children with CKD.

## Materials and methods

### Study design and human research

This is a single-center randomized controlled study. This study was enrolled patients from 2011.1 to 2018.1 by 1:1 and was approved by the Hospital Ethics Committee. The consent of all children was obtained from their guardian. The clinical trial registration number is NO: DSE209931.

### Participants

In this study, 158 children (1–18 years) with vitamin D deficiency and CKD stages of 2–5 were included which were given high- and low-vitamin D supplementation, respectively. The exclusion criteria are receiving vitamin D, growth hormone, or other renal replacement therapy. Children were randomized to receive one of two formulations: standard dose, 400 IU/d of vitamin D, or high dose, 2,000 IU/d. The standard dose was formulated in accordance with vitamin D guidelines of the American Academy of Pediatrics (AAP) (16), and the high dose was formulated to be within the tolerable upper vitamin D intake level specified by the Institute of Medicine (17). Children could only be randomized into the trial for 4 months. Parents of participants received a drop-based formulation to ease the administration of the study drug (Kids Ddrops containing vitamin D3). Informed written consent was obtained from all caregivers, with assent from children, as appropriate. All local research ethics committees approved the studies.

The sample size was estimated at a potential 15% loss rate in 150 patients. The proportions were compared using the type of unilateral test, with a 95% confidence level, 90% statistical power, and 15% accuracy. Subjects were enrolled consecutively from nephrology services. They were continued with their medical, nutritional, and physical protocolized treatments corresponding to their nutritional and CKD statuses provided by their nephrology service, which were not modified by researchers. Acute infection, hospitalization, and refusal to participate were exclusion criteria.

### Randomization and masking

The randomization sequence was generated using a computer-based random number generator by the SickKids research pharmacy. The study biostatistician was unaware of the randomization sequence. Randomization was stratified by practice site with blocks of size 4. The research pharmacy prepared the vitamin D formulations in sealed, serially numbered bottles identical in appearance and weight to maintain allocation concealment. Study personnel, parents, attending physicians, laboratory personnel, investigators, and data analyses were all blinded to group allocation throughout the study period. Research assistants at each site approached participants for entry into the study.

### Procedures and outcomes

**Figure 1:**
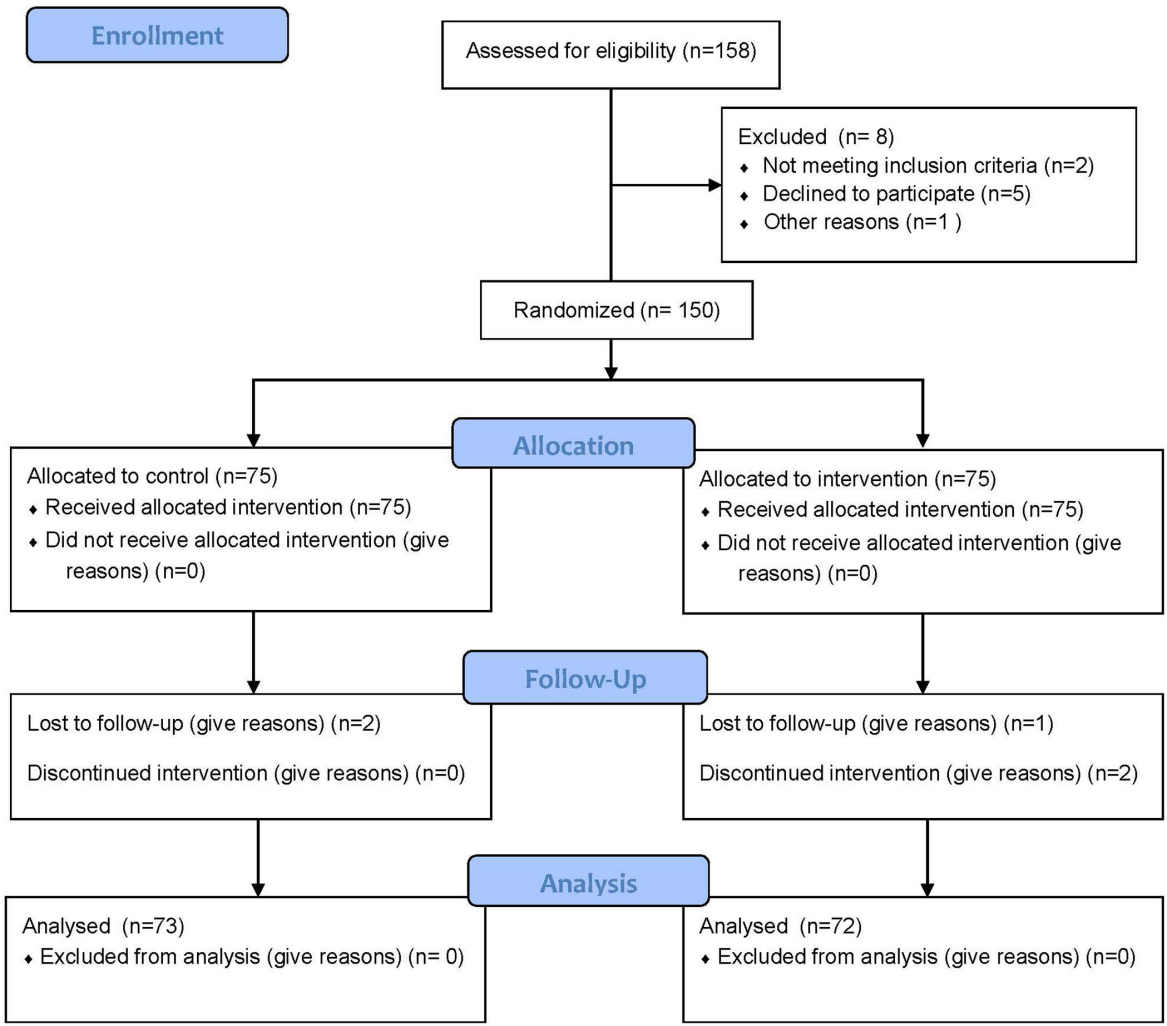
CONSORT 2010 flow diagram.

Figure 1 shows the flowchart of study enrollment. After providing informed consent, parents completed a standardized data collection form, which included age, sex, birth weight, birth height, body mass index, systolic blood pressure (SBP), and diastolic blood pressure (DBP). The mean baseline and 4-month follow-up were compared between the study and control group participants.

Bone mineral density was detected by DXA (GE Medical Instruments), and measurement sites included the lumbar spine 2–4 and femoral neck. CVs that were based on phantom scans ranged from 0.59% (spine) to 5.36% (femoral neck) for BMD. The eGFR was determined by the Schwartz formula using a locally determined k value of 0.33. Vitamin D deficiency was defined as a 25(OH)D <75 nmol/L and vitamin D sufficiency as 75 nmol/L. Hyperphosphatasemia and hyper-/hypocalcemia were defined according to KDOQI guidelines (18). Since vitamin D3 is thought to have equivalent potency, their respective dosages were used to assess associations between vitamin D dosage and other parameters.

Details of laboratory outcome collection, including routine clinical biochemistry tests [serum 25(OH)D, serum calcium, serum phosphorus, magnesium, uric acid, hsCRP, creatinine, albumin, and parathyroid hormone (PTH)] and bone health parameters [specific alkaline phosphatase (ALP), osteocalcin (OC), and procollagen I N-terminal propeptide (PINP)], were measured using validated methodologies.

### Statistical analysis

Data were analyzed on a per-protocol and an intent-to-treat (ITT) basis, with missing data replaced by median values from the opposite group for each primary outcome variable (at each time point) in the ITT analysis. Comparison between the two groups was performed using *t*-tests for normally distributed variables and χ^2^ analyses for categorical data. Multivariable regression analyses were used to investigate covariates including baseline body mass index, eGFR, serum uric acid, hypocalcemia, serum phosphate, serum 25(OH)D, ALP, osteocalcin, spine BMD, and Fummer-BMD. Comparison between two groups was performed using *t*-tests for normally distributed variables and χ^2^ analyses for categorical data. Age, eGFR, serum iPTH, albuminuria, serum uric acid, osteocalcin, and spine BMD were factors associated with final 25(OH)D, and Δ25(OH)D was assessed by Spearman's rank correlations. Data are presented as mean ± standard deviation (SD) or median (25th−75th percentiles) for variables demonstrating parametric and non-parametric distributions, respectively. Statistical significance was determined at a *p* ≤ 0.05. SAS 9.3 (SAS Institute, Cary, NC, USA) or R version 3.3.3 (R Project for Statistical Computing, Vienna, Austria) was used.

## Results

The flowchart of participants is presented in Figure 1. A total of 158 participants were screened, and 150 of them met the inclusion criteria and were randomized. In addition, 145 of them completed all aspects of the 4-month trial. We found no significant difference in age (56 vs. 51 years, *P* = 0.231), BMI (17.32 ± 7.92 kg/m^2^ vs. 18.42 ± 6.23 kg/m^2^, *P* = 0.732), eGFR (49.23 ± 2.93 mL/min/1.73 m^2^ vs. 51.92 ± 5.62 mL/min/1.73 m^2^, *P* = 0.334), serum 25(OH)D (12.92 nmol/L vs. 13.83 nmol/L, *P* = 0.573), and spine BMD (0.731 ± 0.113 g/cm^2^ vs. 0.739 ± 0.098 g/cm^2^, *P* = 0.553) between high- and standard-dose Vit D supplements. The other patients' baseline data are represented in Table 1.

**Table 1:** Baseline demographic, health characteristic, and laboratory values CKD.

**Variable**	**High dose of vitamin D3**	**Standard dose of vitamin D3**	***P*-value**
No. of participants	75	75	
**Demographics**			
Men (%)	56	51	0.231
Age, mean (SD), y	9.2 ± 2.3	10.3 ± 1.9	0.123
Body mass index, mean (SD), kg/m^2^	17.32 ± 7.92	18.42 ± 6.23	0.732
Weight, mean (SD), kg	14.22 ± 2.31	13.92 ± 2.34	0.832
Height, mean (SD), cm	132.22 ± 10.23	136.12 ± 11.34	0.532
Birth weight, mean (SD), kg	17.62 ± 5.34	18.12 ± 6.52	0.346
SBP (mmHg)	115.92 ± 11.23	113.82 ± 8.32	0.122
DBP (mmHg)	63.92 ± 2.35	59.12 ± 7.32	0.324
eGFR (mL/min/1.73 m^2^)	49.23 ± 2.93	51.92 ± 5.62	0.334
hsCRP (mg/L)	9.23 ± 3.11	9.89 ± 1.32	0.452
Serum uric acid (mg/dL)	6.73 ± 1.02	6.02 ± 1.83	0.562
**CKD causes**			
Glomerulonephritis, *n* (%)	14 (18.6)	13 (17.3)	0.109
Cystic kidney disease, *n* (%)	19 (25.3)	21 (28)	0.335
CAKUT (%)	34 (45.3)	31 (41.3)	0.393
Chronic recurrent UTIs associated with reflux	8 (10.6)	10 (13.3)	0.452
Adjusted serum calcium (mmol/L)	2.22 ± 0.12	2.62 ± 0.21	0.552
Hypocalcaemia (%)	9 (12.0)	10 (13.3)	0.632
Serum phosphate (mmol/L)	1.50 ± 0.12	1.73 ± 0.32	0.345
Hyperphosphataemia (%)	19 (25.3)	21 (28.0)	0.123
Serum iPTH (pmol/L)	12.23 (9.21, 23.12)	13.55 (6.23, 19.23)	0.348
Serum 25(OH)D (nmol/L)	12.92 (4.23, 18.12)	13.83 (6.32, 23.23)	0.573
Serum bicarbonate (mmol/L)	21.93 ± 4.23	22.12 ± 3.23	0.632
CRP (mg/L)	0.56 (0.21, 2.01)	0.61 (0.27, 2.66)	0.183
Sclerostin (ng/mL)	0.28 (0.11–0.53)	0.32 (0.23–0.42)	0.732
Alkaline phosphatase (U/L)	510.92 ± 210.23	518.12 ± 183.23	0.328
Osteocalcin (ng/L)	62.92 ± 17.32	69.12 ± 19.23	0.893
PINP (μg/L)	391.92 ± 112.23	434.12 ± 102.32	0.119
Albuminuria (g/mol creatinine)	323 (76, 783)	412 (34, 992)	0.763
Alkaline phosphatase (U/L)	510.92 ± 210.23	518.12 ± 183.23	0.832
Osteocalcin (ng/L)	62.92 ± 17.32	69.12 ± 19.23	0.332
Spine BMD (g/cm^2^)	0.731 ± 0.113	0.739 ± 0.098	0.553
Fummer-BMD (g/cm^2^)	0.783 ± 0.173	0.773 ± 0.121	0.763

CKD, Chronic kidney disease; SBP, Systolic blood pressure; DBP, Diastolic blood pressure; hsCRP, high-sensitivity C-reactive protein; eGFR, glomerular filtration rate; CAKUT, congenital anomalies of the kidney and urinary tract. CPR, C-reactive protein; PINP, procollagen I N-terminal peptide. BMD, bone mineral density.

The primary analysis included 150 participants. We further analyzed the correlation between multiple baseline data. The results showed that the index of chronic kidney disease, such as renal global filtration rate and serum uric acid, was negatively correlated with 25(OH)D levels and BMD. Serum 25(OH)D and osteocalcin levels were positively correlated with spine BMD (Table 2).

**Table 2:** Correlation analysis between kidney and bone metabolism measurements at the high or standard dose of Vit D.

	**Body mass index**	**eGFR**	**Serum uric acid**	**Hypocalcaemia**	**Serum phosphate**	**Serum 25(OH)D**	**ALP**	**Osteocalcin**	**Spine BMD**	**Fummer-BMD**
Body mass index	1									
eGFR	0.32	1								
Serum uric acid	0.31	0.39	1							
Hypocalcaemia	−0.83^*^	0.12	0.34	1						
Serum phosphate	−0.12	0.93	0.11	0.45	1					
Serum 25(OH)D	0.78	−0.32	−0.34^*^	−0.94	0.45	1				
ALP	−0.12	0.21	0.78	0.23	0.78	0.21	1			
Osteocalcin	0.83	−0.88	−0.34^*^	−0.33	0.84	0.22	0.23	1		
Spine BMD	0.28	−0.31	−0.23^*^	−0.11	0.67	0.25^*^	0.53	0.29^**^	1	
Fummer-BMD	0.34	−0.45^*^	−0.83	−0.43	0.43	0.53	0.34	0.35	0.75	1

CKD, chronic kidney disease; SBP, systolic blood pressure; DBP, diastolic blood pressure; hsCRP, high-sensitivity C-reactive protein; eGFR, glomerular filtration rate; CAKUT, congenital anomalies of the kidney and urinary tract; CPR, C-reactive protein; PINP, procollagen I N-terminal peptide; BMD, bone mineral density.

* *p* < 0.05;

** *p* < 0.01;

***p < 0.001.

### Effect of vitamin D supplementation on kidney function

Both high and standard doses of vitamin D improved the serum 25(OH)D level in children, but there was no significant difference in 25(OH)D levels between the two groups. We first analyzed the improvement of the renal global filtration rate and the albuminuria/creatinine ratio of vitamin D supplementation over a 4-week high- and standard-dose treatment. The standard dose of vitamin D can improve serum uric acid levels, but high doses of vitamin D supplementation had no significant effect on serum uric acid. We further compared the effects of high doses and standard doses of vitamin D on kidney function. It was found that high doses of vitamin D were associated with the standard doses of hsCRP and creatinine levels (Table 3). In our study, we first analyzed the effect of high and standard doses of vitamin D on serum 15OHD. The results showed that both high- and normal-dose vitamin D supplementation significantly increased 25OHD levels after the 4-month trial, and the high-dose group showed a more significant increase.

**Table 3:** Changes in biochemical parameters of the high dose and standard dose of vitamin D3.

**Variables**	**High dose of Vitamin D3**			**Standard dose of Vitamin D3**		
	**Baseline**	**4 months**	***p*-value**	**Baseline**	**4 months**	***p*-value**
Serum 25(OH)D (nmol/L)	12.92 (4.23, 18.12)	15.23 (7.23, 27.23)	**0.02**	13.83 (6.32, 23.23)	14.93 (7.23, 24.32)	**0.04**
eGFR (mL/min/1.73 m^2^)	49.23 ± 2.93	59.23 ± 2.31	**0.03**	51.92 ± 5.62	56.92 ± 8.32	**0.01**
hsCRP(mg/L)	9.23 ± 3.11	8.32 ± 1.23	0.33	9.89 ± 1.32	8.89 ± 0.93	0.42
Serum uric acid (mg/dL)	6.73 ± 1.02	5.11 ± 1.02	0.31	6.02 ± 1.83	5.32 ± 2.31	**0.03**
Albuminuria (g/mol creatinine)	323 (76, 783)	283 (52, 673)	**0.011**	412 (34, 992)	335 (53, 732)	**0.013**
Serum phosphate (mmol/L)	1.50 ± 0.12	1.49 ± 0.21	0.832	1.73 ± 0.32	1.62 ± 0.23	0.341
Hyperphosphataemia (%)	19 (25.3)	13 (17.8)	0.138	21 (28.0)	18 (25)	0.329
Serum PTH (pmol/L)	12.23 (9.21, 23.12)	12.93 (8.92, 25.23)	0.877	13.55 (6.23, 19.23)	12.92 (3.23, 21.34)	0.884
Serum calcium (mmol/L)	2.22 ± 0.12	2.52 ± 0.32	0.348	2.62 ± 0.21	2.93 ± 0.42	0.423
Hypocalcaemia (%)	9 (12.0)	4 (5.6)	**0.02**	10 (13.3)	2 (2.7)	**0.031**
Alkaline phosphatase (U/L)	510.92 ± 210.23	419.21 ± 113.23	**0.023**	518.12 ± 183.23	529.22 ± 112.31	0.341
Osteocalcin (ng/L)	62.92 ± 17.32	69.22 ± 11.32	0.126	69.12 ± 19.23	74.92 ± 11.34	0.123
PINP(μg/L)	391.92 ± 112.23	443.92 ± 67.23	0.653	434.12 ± 102.32	402.19 ± 103.88	0.543
Spine BMD (g/cm^2^)	0.731 ± 0.113	0.812 ± 0.034	**0.023**	0.739 ± 0.098	0.883 ± 0.012	**0.032**
Fummer-BMD (g/cm^2^)	0.783 ± 0.173	0.804 ± 0.034	**0.046**	0.773 ± 0.121	0.799 ± 0.073	**0.028**

CKD, chronic kidney disease; SBP, systolic blood pressure; DBP, diastolic blood pressure; hsCRP, high-sensitivity C-reactive protein; eGFR, glomerular filtration rate; CAKUT, congenital anomalies of the kidney and urinary tract; CPR, C-reactive protein; PINP, procollagen I N-terminal peptide; BMD, bone mineral density. The bold values shown the significantly difference between two group.

### Effect of vitamin D supplementation on bone metabolism

Standard and high doses of vitamin D supplementation after 4 months will improve hypocalcemia, spinal bone density, and femur neck bone density. At the same time, high doses of vitamin D supplementation also improve alkaline phosphatase levels. When comparing the results of different doses of vitamin D supplementation, it was found that high-dose vitamin D supplementation did not improve bone density in the spine and femur neck relative to the standard dose of vitamin D but improved hypocalcemia and PINP levels (Table 4).

**Table 4:** Predictors of final 25(OH)D levels and changes in serum concentrations of 25(OH)D during vitamin D supplementation; results of multiple linear regression analyses.

**Outcome**	**Predictor**	**β (standard error)**	***P*-value**
Final 25(OH)D	Age	−3.732 (0.231)	0.023
	eGFR	−5.231 (0.832)	0.012
	Serum iPTH	−0.563 (0.032)	0.041
	Albuminuria	−21.23 (1.341)	0.023
	Serum uric acid	−3.21 (0.233)	0.011
	Osteocalcin	73.23 (10.332)	0.001
	Spine BMD	3.23 (0.342)	0.031
Δ25(OH)D	eGFR	−3.529 (0.732)	0.042
	Serum calcium	2.31 (0.232)	0.023

CKD, chronic kidney disease; SBP, systolic blood pressure; DBP, diastolic blood pressure; hsCRP, high-sensitivity C-reactive protein; eGFR, glomerular filtration rate; CAKUT, congenital anomalies of the kidney and urinary tract; CPR, C-reactive protein; PINP, procollagen I N-terminal peptide; BMD, bone mineral density.

## Discussion

Chronic kidney disease in children often leads to abnormal bone metabolism, which, in turn, affects the health of the bone system. Our study analyzed the effects of different doses of vitamin D on kidney function and bone metabolism in children with chronic kidney disease and found that vitamin D supplementation improved kidney and bone metabolism abnormalities. Furthermore, compared with the standard dose of vitamin D, the high dose of vitamin D supplementation showed improvement in kidney function and alkaline phosphatase levels, but no significantly difference in bone density was found.

Bone metabolism in children with chronic kidney disease is a triad of biochemical imbalances of calcium, phosphate, parathyroid hormone, vitamin D, bone abnormalities, and soft tissue calcification. Ewert et al. found that patients with nephropathic cystinosis had severe skeletal comorbidity associated with distinct CKD stage-dependent alterations of bone metabolism than CKD controls, suggesting impaired mineralization and increased bone resorption, which are only partially normalized after renal transplantation (19). Huang et al. investigated the higher prevalence of hip fracture, relative to that of the spine, among patients with CKD and generated meaningful insights to guide care, prevention, and treatment regimens for patients with CKD (12). Our analysis also found a significant correlation between kidney disease indicators and bone metabolism-related indicators.

Vitamin D deficiency (<20 ng/mL) and insufficiency (20–29 ng/mL) are common among patients with chronic kidney disease (CKD) or those undergoing dialysis (20). Vitamin D supplements may play important roles in CKD. Gluba-Brzózka A et al. found that multiple observational studies have demonstrated an association between the use of active vitamin D therapy in patients on dialysis and those with CKD, and improved survival (21). A meta-analysis found that the use of vitamin D supplements, especially vitamin D3, could reduce the incidence of falls. Only vitamin D with calcium supplement showed benefit in fracture reduction (22). For patients with CKD stages 3–4 and vitamin D deficiency, vitamin D supplementation may improve vascular function (23). However, compared with placebo, vitamin D3 supplementation resulted in no significant difference in change in the eGFR at 5 years among adults with type 2 diabetes (24). This is also consistent with our finding that vitamin D supplementation also improves kidney function. New evidence has established a new paradigm in the management of patients with CKD having vitamin D deficiency, and it appears in some studies that adequate replacement of vitamin D in the deficient population can reduce premature mortality and morbidity in the CKD population (25). Ghosh and Ghosh found that vitamin D deficiency was more pronounced in advanced stages of CKD, and the eGFR was strongly associated with serum vitamin D levels (25). Teumer et al. found that circulating vitamin D metabolite levels are negatively associated with the eGFR (26). In our study, the eGFR had a negative correlation with 25(OH)D levels. However, it is worth noting that the relationship between vitamin D supplementation and the eGFR is not so clear in the presence of differences in baseline vitamin D levels (27).

Vitamin D deficiency is also associated with many diseases, such as enhancing the risk of osteoporotic fractures. Randomized controlled trials in children and adolescents are urgently needed to support the potential of vitamin D as a complementary therapeutic option in mental disorders (28). For breastfed infants, a vitamin D supplementation of 400 IU/day for up to 6 months increases 25-OH vitamin D levels and reduces vitamin D insufficiency, but insufficient evidence exists to assess its effect on vitamin D deficiency and bone health. For high-risk infants who are being breastfed, maternal vitamin D supplementation reduces vitamin D insufficiency and vitamin D deficiency, but insufficient evidence exists to determine its effect on bone health (29). A spontaneous decrease in the GFR reduces the effect of vitamin D on the improvement of renal function. A spontaneous GFR decrease is mostly seen due to primary glomerular diseases and secondary kidney damage. (1) The most common primary glomerular diseases include IgA nephropathy, thylakoid proliferative glomerulonephritis, membranoproliferative glomerulonephritis, and membranous nephropathy, all of which can damage the filtration system of the kidney, resulting in reduced glomerular filtration rates. (2) The most common secondary kidney damage include diabetic nephropathy, hypertensive kidney damage, lupus nephritis, purpura nephritis, and hepatitis B-related nephritis. If these diseases are not actively treated, they will affect the glomerular filtration function and lead to a reduced glomerular filtration rate (30–34).

Although it is clear that vitamin D supplementation can significantly help improve bone metabolism, whether high doses of vitamin D supplementation have a positive effect on the skeletal system is still controversial. Burt et al. investigated that among healthy adults, treatment with vitamin D for 3 years at a dose of 4,000 IU per day or 10,000 IU per day resulted in statistically significant lower radial BMD than treatment with 400 IU per day; tibial BMD was significantly lower only with the 10 000 IU per day dose. No significant differences in bone strength were identified at either the radius or tibia. These findings do not support the advantage of high-dose vitamin D supplementation for bone health (15, 35). Our results show a significant correlation between serum vitamin D levels and bone mass, but vitamin D supplementation can not further improving bone mass. In our study, high doses of vitamin D improved hypocalcemia and PINP. The possible reason is related to the promotion of calcium absorption. Gorman et al. also found that high-dose intramuscular vitamin D provides long-lasting moderate increases in the serum 25-hydroxvitamin D level and short-term changes in the plasma calcium level (36). In addition, previous studies found that vitamin D intake over the recommended dose is often associated with a high serum 25(OH)D level and mostly not associated with symptoms of hypercalcemia (37). A sandwich enzyme-linked immunosorbent assay (ELISA) for quantification of the N-terminal propeptide of human procollagen type I (PINP) utilizing purified alpha 1-chain specific rabbit antibodies is described (38). High doses of vitamin D are also associated with an increased in PINP (39).

In our study, we further analyzed the effects of different doses of vitamin D on the bone system of children with chronic kidney disease. We found that the high dose of vitamin D in the bone system has no significant effect on bone metabolism in chronic kidney disease. This is also consistent with previous study results. Lauren et al. found that high-dose vitamin D supplementation cannot improve bone health in adults (15). Furthermore, for adult women, a previous study has shown that high doses of vitamin D have a greater adverse effect on the volumetric bone density (35). High doses of vitamin D also have a significant effect on kidney indicators. Thierry et al. found a small (−15%) but significant decrease in albuminuria after high-dose vitamin D supplementation (40). A similar result has been found in our results. In animal experiments, Wang et al. showed that exposure to a high dose of vitamin D3 decreased the levels of serum creatine, urea nitrogen, and urine protein and restored the homeostasis of calcium and parathormone, and vitamin D3 acts as a potential antifibrotic drug in chronic kidney disease *via* the vitamin D receptor and inhibits the TGF-β1/Smad3 signaling pathway (41). High-dose vitamin D supplementation can reduce the prevalence of premenstrual syndrome and dysmenorrhea as well as has positive effects on the physical and psychological symptoms of premenstrual syndrome (42). The improvement in the lumbar spine bone mineral density z-score was more enhanced with high-dose VitD/Ca supplementation than the standard dose (39). However, Lippi et al. concluded that treatment of healthy adults with highdose vitamin D supplementation for 3 years did not have any beneficial effect on volumetric bone mineral density (BMD) and bone strength (43). Further studies are needed on the effect of different doses of vitamin D on the improvement of bone mass and renal function.

In our study, children were randomized to receive 1 of 2 formulations: the standard dose, 400 IU/d of vitamin D, or the high dose, 2,000 IU/d. The standard dose was formulated in accordance with vitamin D guidelines of the American Academy of Pediatrics (AAP) (16), and the high dose was formulated to be within the tolerable upper vitamin D intake specified by the Institute of Medicine (17). In addition, calcidiol or calcitriol and calcium-based phosphate binders are important in the treatment of osteoporosis, thus, in our future studies, we will include the effect of the same calcidiol or calcitriol and calcium-based phosphate binders and other treatment modalities on bone metabolism in children with kidney disease.

There are several limitations to this study. First, this was a single-center RCT study. Second, five patients were discharged within 4 months. Finally, 145 patients were analyzed in our study. The modest sample size could have affected the accuracy of the analysis. In addition, the use of DXA in our CKD pediatric group is limited due to the unique growth and stature of our patients. Future studies should include multiple centers with larger sample sizes to evaluate the efficacy of the different doses. Finally, bone turnover comprises two processes: the removal of an old bone (resorption) and the laying down of a new bone (formation). The N-terminal propeptide of type I procollagen (PINP) and C-telopeptide of type I collagen (CTX-I) are markers of bone formation and resorption, respectively, that are recommended for clinical use. Bone turnover markers can be measured on several occasions in one individual with good precision (3, 4). In our study, we did analyze only osteogenesis-related metrics. We used bone imaging metrics to clarify bone mass changes, while osteogenic metrics were used to demonstrate the effect of vitamin D on bone formation. We assume this is important to confirm the effect of vitamin D on bone mass improvement. In our future studies, we will include further bone resorption markers to analyze the effect of vitamin D on bone resorption.

## Conclusion

Among the children with clinical kidney disease, treatment with vitamin D for 4 months at the high-dose vitamin D treatment resulted in statistically significantly improved kidney function but no significantly different bone metabolism compared with the standard-dose treatment.

## Data availability statement

The original contributions presented in the study are included in the article/supplementary material, further inquiries can be directed to the corresponding authors.

## Ethics statement

This study was approved by Ethics Committee of Shandong First Medical University (NCSD2031233). The patients/participants provided their written informed consent to participate in this study.

## Author contributions

YF, KL, HL, and YM: guarantor of integrity of the entire study. YM, FL, and XZ: study concepts. ZF, YM, FL, XZ, YF, and KL: study design, experimental studies, data acquisition, data analysis, statistical analysis, and manuscript review. ZF, YM, and FL: definition of intellectual content, literature research, and clinical studies. XZ and YF: manuscript preparation and manuscript editing. All authors contributed to the article and approved the submitted version.

## Funding

This research was funded by the Academic Promotion Program of Shandong First Medical University (Grant No. 2019QL017). This work were carried in The Second Affiliated Hospital of Shandong First Medical University.

## Conflict of interest

The authors declare that the research was conducted in the absence of any commercial or financial relationships that could be construed as a potential conflict of interest.

## Publisher's note

All claims expressed in this article are solely those of the authors and do not necessarily represent those of their affiliated organizations, or those of the publisher, the editors and the reviewers. Any product that may be evaluated in this article, or claim that may be made by its manufacturer, is not guaranteed or endorsed by the publisher.
